# Nectar Theft and Floral Ant-Repellence: A Link between Nectar Volume and Ant-Repellent Traits?

**DOI:** 10.1371/journal.pone.0043869

**Published:** 2012-08-29

**Authors:** Gavin Ballantyne, Pat Willmer

**Affiliations:** University of St Andrews School of Biology, St Andrews, Fife, United Kingdom; University of Northampton, United Kingdom

## Abstract

As flower visitors, ants rarely benefit a plant. They are poor pollinators, and can also disrupt pollination by deterring other flower visitors, or by stealing nectar. Some plant species therefore possess floral ant-repelling traits. But why do particular species have such traits when others do not? In a dry forest in Costa Rica, of 49 plant species around a third were ant-repellent at very close proximity to a common generalist ant species, usually via repellent pollen. Repellence was positively correlated with the presence of large nectar volumes. Repellent traits affected ant species differently, some influencing the behaviour of just a few species and others producing more generalised ant-repellence. Our results suggest that ant-repellent floral traits may often not be pleiotropic, but instead could have been selected for as a defence against ant thieves in plant species that invest in large volumes of nectar. This conclusion highlights to the importance of research into the cost of nectar production in future studies into ant-flower interactions.

## Introduction

Ants are capable of disrupting pollination when visiting flowers (e.g., [1], [2], [3]). How often do plant traits prevent this from happening? When feeding on other plant surfaces ants can benefit a plant (e.g., preying upon herbivores, or disrupting their feeding and oviposition), whilst ants attracted by hemipteran honeydew may reduce levels of more damaging herbivores [4], [5]. Many plant species produce extrafloral nectar (EFN), which actively attracts ants, and which may be sited to encourage ants to patrol vulnerable areas such as new growth [6] and inflorescences [7], [8]. In tropical canopies plant exudates from hemipteran honeydew and EFN can support huge populations of potentially beneficial ants [9], [10], [11]. Some myrmecophytes also provide housing (domatia) for ant colonies, as hollow stems or thorns, and thereby acquire standing armies of specialized mutualists that they may feed with EFN, protein bodies, or indirectly with hemipteran honeydew [12], [13], [14]. These specialized ants may provide additional benefits by pruning encroaching vegetation (e.g., [15]), or supplying nutrients to their hosts through detritus within domatia (e.g., [16]).

However, there are some situations where attracting ant-guards may be detrimental, most obviously during flowering. Being flightless, ants are generally ineffective as pollinators themselves and may also reduce pollen viability due to the antibiotic secretions used in nest hygiene [17], [18]. Additionally they may reduce visitation and disrupt pollination by stealing nectar or threatening incoming pollinators (e.g., [2], [19], [20]), resulting in decreased seed set [1]; this is especially damaging where plants are self-incompatible and rely on limited supplies of outcrossed pollen [21]. Ant attendance at flowers can therefore reduce plant fitness (e.g., [3], [22], [23]), and are only encouraged onto inflorescences by EFNs where their anti-herbivore benefits outweigh possible costs.

Perhaps for this reason, ants are repelled from certain flower species in response to contact with mechanical barriers such as trichomes, or through chemical deterrents including flower scent [24], [25]. Thus, obligate potential flower visitors may be attracted to floral scents while facultative visitors such as ants may be repelled [26]. Most work on ant-floral conflict has focused on *Acacia*, both in Africa and the Neotropics, where repellence by floral scent has been demonstrated [27], [28], [29], [30]; in these cases volatile organic chemicals (VOCs) released by young *Acacia* inflorescences repel the resident ant-guards for a few hours, allowing pollinators free access. Some VOCs from pollen may mimic ant alarm pheromones, and these VOCs peak in the myrmecophtye *A. seyal fistula* during dehiscence [30]. Other temperate and tropical plant species have also been found to possess VOC ant-repellence [30], [31], [32], [33], [34].

In most cases to date this repellence involves a pollen- or anther-derived scent, transferable though “pollen-wiping” (e.g., [27], [35]). As yet, however, the range of plant species studied is small and the causes of repellence poorly understood. Where ant-repellence has been identified in non-ant-plants its function is less obvious, as the costs and benefits of ant attendance at flowers have only rarely been tested (e.g., [2], [3], [20]). Is ant repellence selected to reduce aggression towards pollinators, or to prevent nectar theft, or is it due to pleiotropic effects on other floral traits? This study aimed to identify patterns of occurrence of repellence in the following ways:

1. Which plant species possess ant-repellent traits and what form do those traits take: are they effective over a long range or do ants have to contact the flowers?
2. What plant and floral traits are correlated with floral ant-repellence? If repellence is adaptive it may be commoner in species investing most heavily in flowers (e.g., in advertisements such as copious pollen or large nectar volumes), and less common in plant species that limit access to rewards through morphological traits. If there is a relationship between rewards and/or accessibility then repellence may also relate to phylogeny and/or to pollination syndrome.
3. How effective are ant-repellent traits against different functional groups of ant species? If such traits function to reduce threats to pollinators, they might be selectively targeted at larger predatory ants.

Better understanding of the types of plants that restrict ant access to flowers will give insight into the potential selective pressures structuring interactions between ants and plants at this crucial stage in the plant life cycle.

## Methods

### a) Ethics Statement

In 2008 field work was carried out in Costa Rica at La Selva and Las Cruces, operated by the Organisation for Tropical Studies (permit issued by Javier Guevara, ref: 045-2008-SINAC). In 2009 and 2010 field work was carried out in Costa Rica at Santa Rosa National Park, operated by the Area de *Conservación* Guanacaste (permits issued by Roger Blanco, ref: ACG-PI-007-2009 and ACG-PI-003-2010). All fieldwork was carried out at these sites and during the periods covered by these permits.

### b) Study sites

Fieldwork was carried out in Costa Rica initially in tropical wet forest at La Selva Biological Station (10°26′N, 83°59′W) and Wilson Botanical Garden at Las Cruces (8°47′N, 82°57′W) from February to May of 2008. Further data were gathered in tropical dry forest at Santa Rosa National Park in North-western Costa Rica (10°54′N, 85°39′W) from January to May of 2009 and January to February of 2010. These sites provided both logistical support and a broad diversity of plant species. The dry forest was structurally ideal for accessing and observing a wide variety of flowers and ants are commonly found on all plant species.

### c) Study species

To test for evidence of ant-repelling traits, plant species were chosen with a wide variety of floral forms and from a range of taxonomic groups, but selection was limited by availability. Plants were initially watched from dawn to identify the period of peak dehiscence; some species had pollen available all morning but others only in the short window after dehiscence, before all pollen was lost to visiting bees. Flowers were thereafter collected at times of high pollen availability.

The generalist ant *Camponotus novograndensis* (Formicinae) was used for the majority of experiments. It is a regular visitor to EFNs and flowers and was especially common at Santa Rosa. Other ant species were chosen that were common and that represented various feeding preferences and taxonomic groups. The most important of these secondary ant species was the large *Ectatomma ruidum* (Ectatomminae), which does feed from EFNs and flowers but, unlike *C. novograndensis*, has also been shown to hunt bees [36]. Workers of average size for each ant species were used in all experiments and collected from the same nest in each study area. Hence, all ants used for a single plant species were nestmates. Each ant was collected by allowing passive entry into a glass tube from ground or vegetation, was used once, and then returned to the area where found (except for a few collected for identification).

### d) Detecting the presence and form of ant-repellent traits

**i. Tactile response trials** identified repellence when an ant contacted part of a flower. A single ant was placed in a Petri dish with three or four evenly spaced objects: a dehisced flower, an older pollen-depleted flower and/or a bud (as available from that species), and an appropriately-sized twig used as a control to rule out neophobic responses to unfamiliar objects. Various behaviours were recorded over four minutes: the number of antennations, the times an object was walked over or clearly avoided, and probing of the objects. Repellence was deemed to occur when an ant's antennae contacted an object and the ant immediately jerked or turned away (often followed by grooming of the antennae). Recording began 10 s after an ant was placed in the arena, reducing the likelihood of recording purely neophobic responses. At least 12 trials were carried out for each species combination of ant and plant (unless fresh flowers became unavailable). We predicted negligible ant-repellence from buds and pollen-free older flowers but had no prior expectation of ant-repellence for specific non-ant plants. The upper petal surfaces of fresh flowers of *Stachytarpheta jamaicensis* (regardless of pollen availability) were strongly ant-repellent, so we tested these flowers in further tactile response trials using *C. novograndensis.* Fresh petals were rubbed against 1 cm^2^ of filter paper with untouched paper for controls.

Tactile response data were analysed using the number of times that an ant walked over or were repelled by floral parts within a single trial. Although other behaviours were recorded, repellence from an object provided a clear, unambiguous response. Proportions repelled were calculated following Junker et al. [31]: ‘0’ = never repelled on contact, ‘1’ = always repelled on contact. An ant walking over just the base of a corolla was not included in the proportion data; and ants “repelled” by the twig “control” were discounted from the analysis entirely, though in practice this only excluded a very small number of trials. Floral parts for trials were picked with clean forceps and used immediately, and only once. Equipment was washed carefully with alcohol between trials.

**ii. Scent response trials** tested longer range (non-contact) repellence, arising from VOCs. An ant was placed in a Petri dish connected to two syringes, one empty and acting as a control (for air movement alone) and the second containing a flower or inflorescence. (Although a larger number of flowers could have been used to concentrate the scent we decided to use scents at concentrations close to what an ant would experience when approaching a flower.) Ant behaviour was observed for 4 min alone inside the arena, 1 min after control air was gently blown through, and for another 1 min after this was repeated with the flower scent. The number of times an ant changed direction (a turn of more than 90°) and/or crossed to the centre of the arena was recorded as a proxy for increased activity. Agitation or aggression were recorded as “charges”, “abdomen cocks”, “holding the head up” and “time spent grooming”. Time spent stationary was also recorded. If ants responded in any way to floral scent, trials were repeated with just vegetative plant parts to confirm that the VOCs responsible originated from floral tissue.

### e) Detecting plant and floral traits correlated with floral ant-repellence

Scores were assigned to each plant species for nectar volume and nectar accessibility. Mean nectar volume at dehiscence was scored categorically: 0 = no nectar detectable, 1 = volume too small to collect with a 1 µl microcap, 2 = <0.5 µl, 3 = >0.5 µl. Nectar accessibility to ants was scored by flower shape from 1 (flowers with very limited access to nectar) to 4 (open access to nectar for ants of all sizes), taking into account width of the corolla and obstructing trichomes or anthers. Each plant species was defined as specialised or generalised (cf., [37], [38], [39] for detailed discussions of these terms) depending on the pollination syndrome to which the floral traits appeared to conform [39], and/or its known visitors.

### f) Effectiveness of ant-repellent traits against different ant species

Plant species found to possess effective ant-repellent traits in tactile and/or scent trials with *C. novograndensis* were tested against other ant species. 14 species were tested against *Ectatomma ruidum* in tactile trials, and 11 species in scent trials (10 species from initial trials at La Selva and a single species, *Randia monantha*, at Santa Rosa). Five plant species with very different floral forms that were highly repellent to *C. novograndensis* were also tested against an additional 6–7 ant species with very different behavioral habits (detailed in Table 1). To test for repellence in another example from the genus *Stachytarpheta*, the ant species *Acromyrmex coronatus*, *Crematogaster curvispinosa, Ectatomma ruidum*, *Megalomyrmex foreli* and *Pheidole fallax* were also tested in tactile trials against *Stachytarpheta frantzii*.

**Table 1:** Summary of behavioral trials carried out to address each experimental question.

Experimental Questions	Method	Plant Species	Ant Species
1 and 2	Scent Trials	33 plant species (see Table S1)	*Camponotus novograndensis* (Formicinae) [medium sized generalist]
	Tactile Trials	49 plant species (see Figure 1 and Table S1)	*Camponotus novograndensis* (Formicinae) [medium sized generalist]
3	Scent Trials	11 plant species	*Ectatomma ruidum* (Ectatomminae) [large predator]
	Tactile Trials	14 plant species (see Figure 3)	*Ectatomma ruidum* (Ectatomminae) [large predator]
		*Barleria oenotheroides* (Acanthaceae) [under-canopy herb]; *Cordia alliodora* (Boraginaceae) [myrmecophytic tree]; *Malvaviscus arboreus* (Malvaceae) [shrub]; *Ruellia inudata* (Acanthaceae) [herb with defensive trichomes]; *Stachytarpheta jamaicensis* (Verbenaceae) [EFN-bearing herb]	*Atta cephalotes* (Myrmecinae) [leafcutter]; *Camponotus sericeiventris* (Formicinae) [large generalist]; *Cephalotes umbraculatus* (Myrmecinae) [arboreal]; *Pachycondyla villosa* (Ponerinae) [large predator]; *Pheidole fallax* (Myrmecinae) [small generalist]; *Pseudomyrmex gracilis* (Pseudomyrmecinae) [medium-sized generalist]; *Cephalotes setulifer* (Myrmecinae) [*C. alliodora* plant-ant, only tested with *C. alliodora*]

### g) Statistical methods

As data were not normally distributed and data transformations were not possible Kruskal Wallis tests were used to compare the proportions of ants repelled from floral parts within species and to identify differences between proportion of ants repelled and floral traits of the various species used. As 49 tests were carried out a Bonferroni correction was applied to the significance level. Jonckheere-Terpstra tests were used to explore correlations among these groups. Behaviours recorded during the control and test minutes of the scent trials were compared using Kruskal Wallis tests. All statistical tests were carried out using SPSS 18.0.

## Results

### a) Which plant species possess ant-repellent traits and what form do they take?

**Figure 1:**
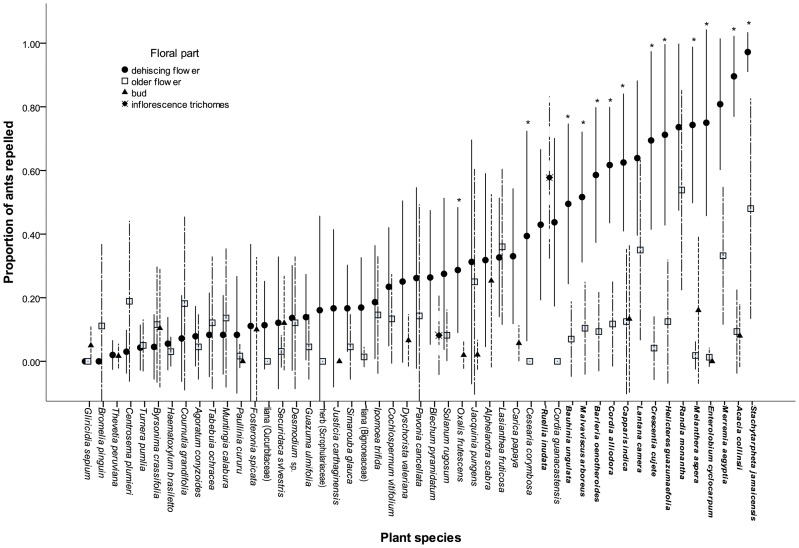
Tactile response results showing the mean proportion of *Camponotus novograndensis* workers repelled by the fresh dehiscing floral parts and control parts of each plant species. Results arranged by increasing repellence from fresh flowers. In the case of *Ruellia inudata* and *Blechum pyramidatum* areas of inflorescence carrying trichomes were used. Floral parts of species in bold repelled ants on more than 50% of encounters and those marked * repelled ants significantly more from fresh flowers than control parts used (pollen-depleted flowers or buds). At least 12 replicates were carried out for each plant species. Error bars indicate 95% confidence intervals.

We used *Camponotus novograndensis* in tactile trials with 49 plant species and observed a wide range of responses (Figure 1). The fresh flowers of 15 species (bold in Figure 1) repelled ants on more than half of the occasions that ants touched them. In 12 of these highly repellent species and in 4 that were less repellent (* in Figure 1), fresh dehiscing flowers were significantly more repellent than the control objects tested (older flowers with pollen-depleted anthers or unopened buds) (results of K-W tests shown in Table S1). In most cases repellence involved contact with pollen-bearing anthers, with the exception of *Ruellia inudata* and *Merremia aegyptia*, where trichomes on the inflorescence were entirely or partly responsible respectively, and *Stachytarpheta jamaicensis*, where the upper petal surface was repellent. Ants would occasionally walk over the bases of flowers with repellent pollen but would avoid the stamens.

Scent trials identified only the shrub *Randia monantha* as having a floral odour that influenced *C. novograndensis*, with a significant increase in agitated behaviour following injection of floral scent into the arena (χ^2^ = 5.3, df = 1, p = 0.021). There was no increase in agitation with VOCs from a cut stem of the plant, i.e. the effect was specific to floral VOCs.

### b) Which plant and floral traits are correlated with floral ant-repellence?

**Figure 2:**
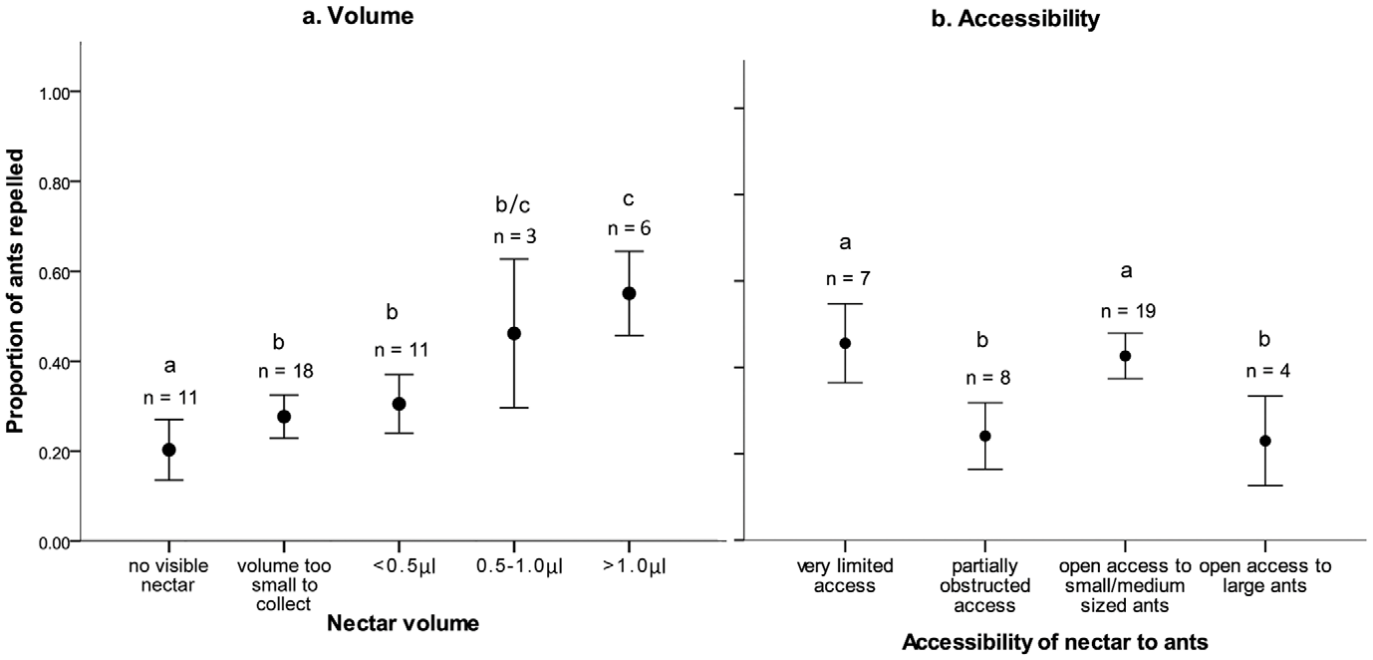
Tactile ant-repellence, nectar volume and nectar accessibility. a) Significant differences in ant-repellence occurred between plants with different volumes of nectar (χ^2^ = 42.9, df = 4, p<0.001) with a significant positive trend between nectar volume and the proportion of ants repelled (J-T = 5.9, df = 4, p<0.001). b) Ant-repellence differed significantly between plants with different levels of accessibility to nectar (χ^2^ = 26.6, df = 3, p<0.001), although the negative trend between accessibility and repellence was not significant (J-T = −1.5, df = 3, p = 0.13). Significant differences between groups indicated with a/b/c. Error bars indicate 95% confidence intervals.

There was a significant positive relationship between the degree of floral ant-repellence and nectar volume (Figure 2a). Plants producing large nectar volumes were more likely to have protection against nectar theft. There were also significant differences between flowers with varying accessibility to nectar for ants (Figure 2b); however, there was no overall significant trend.

There was no significant difference in repellence between species with flowers exhibiting specialised pollination syndromes and more generalised species (χ^2^ = 0.702, df = 1, p = 0.402). However, possibly due to the large nectar volumes required to attract endothermic vertebrates [39], [40] flowers showing bird- and bat-pollination traits were significantly more ant-repellent than other flowers (χ^2^ = 36.654, df = 1, p<0.001). The proportions of ants repelled from flowers of species with different growth forms were significantly different (χ^2^ = 11.2, df = 3, p = 0.011), but this was entirely due to the effect of climbers (no difference when excluded (χ^2^ = 2.6, df = 2, p = 0.28)).

The proportion of ants repelled varied significantly between plant families from which more than one species was tested (χ^2^ = 30.236, df = 8, p<0.001). However, the two best surveyed families, Acanthaceae (n = 6) and Fabaceae (n = 8), did not significantly differ in ant-repellence (χ^2^ = 0.989, df = 1, p = 0.32) suggesting that between-family variation in ant-repellence may to be an artefact of low sampling sizes within families.

### c) How effective are ant-repellent traits against differing ant species?

**Figure 3:**
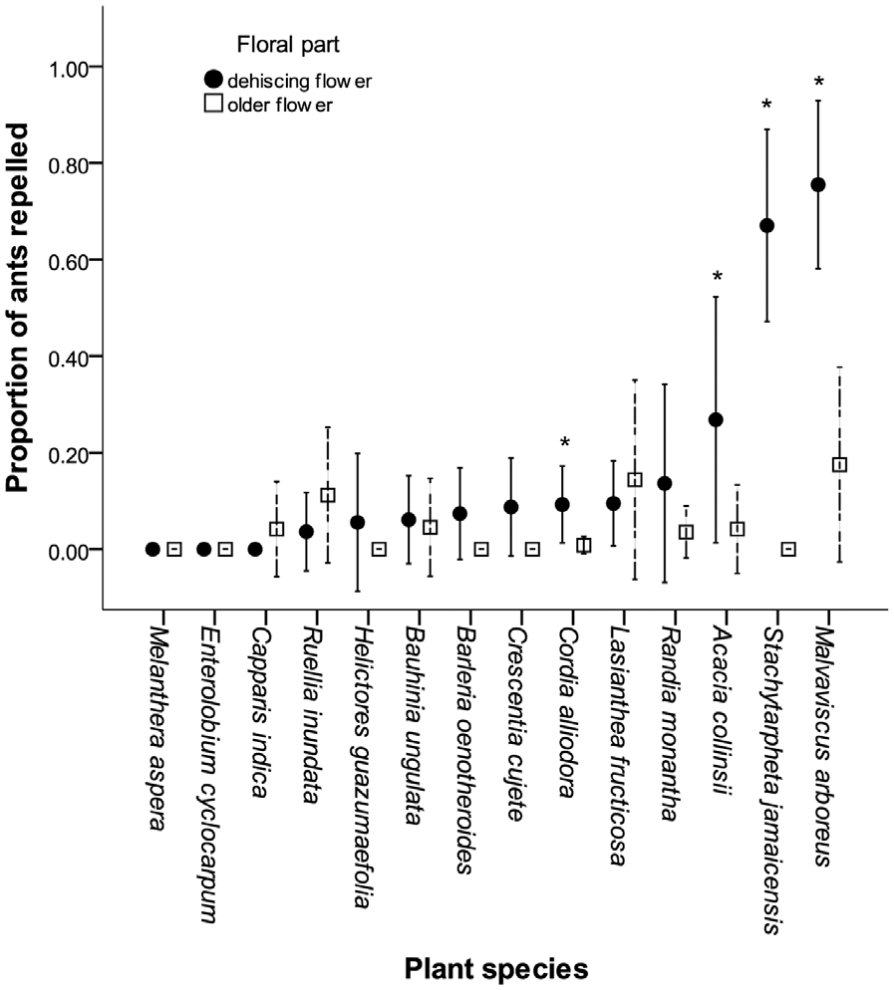
Plant species tested for tactile ant repellence with *Ectatomma ruidum*. * indicates significant difference between freshly dehisced flowers and old pollen-depleted flowers. Error bars indicate 95% confidence intervals.

**Figure 4:**
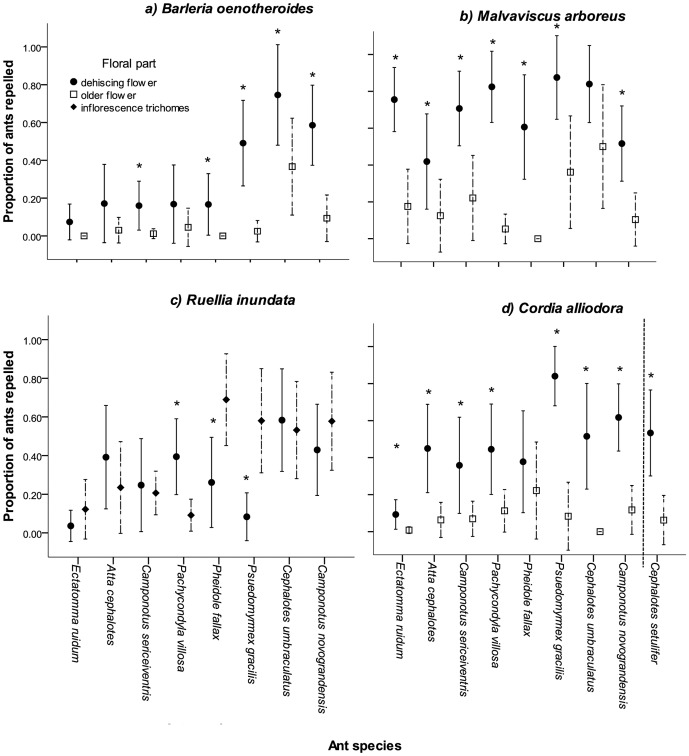
Floral ant-repellence in flowers tested against a range of ant species (including *Cephalotes setulifer* tested only with its host *C. alliodora*). (a) *Barleria oenotheroides*, (b) *Malvaviscus arboreus,* (c) *Ruellia inudata* and (d) *Cordia alliodora.* * indicates significant difference between freshly dehisced flowers and old pollen-depleted flowers. Error bars indicate 95% confidence intervals.

Of the plant species that repelled *C. novograndensis* in the tactile trials, 14 were also tested against *Ectatomma ruidum* (Figure 3), but only two had a significant repellent effect (the fresh anthers of *Malvaviscus arboreus,* and the upper petal surface of *Stachytarpheta jamaicensis*). *Stachytarpheta frantzii,* tested in 2008, had also provoked a repellent response from *E. ruidum*. In scent trials *Randia monantha* flowers had no effect on *E. ruidum* behaviour, and the scent trials carried out with *E. ruidum* at La Selva with 10 other plant species also found no repellence. Figure 4 (a–d) shows results with 4 plant species tested with multiple ant species.

- As well as repelling *Camponotus novograndensis*, the fresh pollen of *Barleria oenotheroides* strongly repelled two other ants, *Cephalotes umbraculatus* and *Pseudomyrmex gracilis* (Figure 4a), both being species small enough to raid for nectar (although whether the normally arboreal *C. umbraculatus* forages on such low-lying vegetation is unknown). All other ant species tested were not repelled by *B. oenotheroides* and indeed often walked over pollen-laden anthers.
- *Ruellia inudata* flowers were only repellent to *C. novograndensis* and *C. umbraculatus* (Figure 4b). This plant's inflorescences below the flowers were covered in hairs, but these were only repellent to ant species with shorter legs.
- *Cordia alliodora* pollen possessed a more generalised ant-repellence, affecting all ants tested except *E. ruidum* (Figure 4c) and the three ant species most strongly repelled were those most common on the tree at Santa Rosa.
- *Malvaviscus arboreus* pollen provoked a strong repellent response from all the ant species tested (Figure 4d).

**Figure 5:**
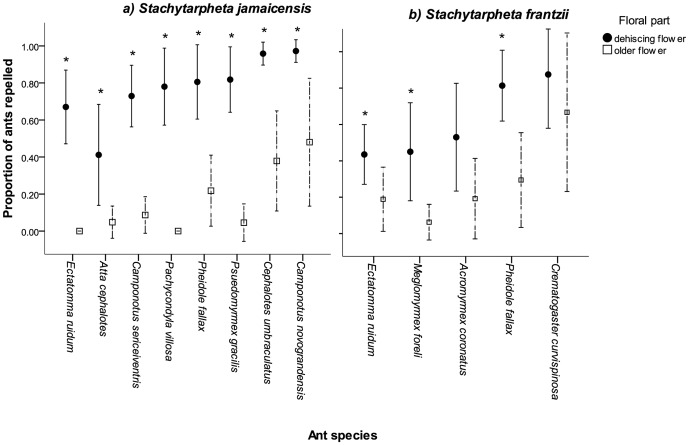
Floral ant-repellence in two species of *Stachytarpheta* tested against different ant species. (a) *Stachytarpheta jamaicensis* and (b) *S. frantzii.* * indicates significant difference between freshly dehisced flowers and old pollen-depleted flowers. Error bars indicate 95% confidence intervals.

Figure 5 shows effects with both species of *Stachytarpheta* studied, which possessed strong, generalised ant-repellence elicited from the upper petal surface of fresh flowers. *S. jamaicensis* had the most potent general ant-repellence (Figure 5a). *Ectatomma ruidum* was repelled less by the petals of *S. frantzii* than by those of *S. jamaicensis* but there was no significant difference in the responses of *Pheidole fallax,* (∼80% repelled) (Figure 5b). The tactile response trials for *S. jamaicensi*s wiped on filter paper showed no significant difference between the proportion of ants repelled by test and control pieces (average proportion repelled = 0.125; χ^2^ = 0.52, df = 1, p = 0.47), so the repellence was not easily transferable to another surface.

Clearly ant species responded to flowers very differently. As the ant *Cephalotes umbraculatus* was moderately repelled by floral parts of all flower species tested, additional tactile trials were carried out using the flowers of *Cornutia grandiflora* (which had not proved repellent to *Camponotus novograndensis*), to ensure that it did not have a generic response to all flowers. *C. umbraculatus* was not repelled (average proportion repelled = 0.07; χ^2^ = 0.68, df = 1, p = 0.41). At the other extreme, we found no particularly strong repellent effect of any flowers on *Atta cephalotes.* This species is not a nectar thief, and furthermore as these leafcutters can gain access to flower bases, it is unlikely that anther-based ant-repellence would provide any defence against them.

## Discussion

### a) Ant-repellence - protecting an investment in nectar?

In this study floral ant-repellence was observed most frequently in plant species producing high volumes of nectar per flower. Avoidance of nectar theft may therefore have influenced floral evolution, with selection for ant-repelling floral traits to protect against species (such as *Camponotus novograndensis*) that commonly visit flowers and steal nectar without providing any beneficial pollination services. In nearly all species tested here, individual flowers lasted for just a single day, and pollen was usually available for only a few hours after sunrise. With such a narrow window for male reproductive success, large numbers of ants thieving nectar from a flower could significantly reduce fitness. By repelling the initial forays of scouting workers, the plant prevents recruitment of large numbers of additional ants to valuable sources of sugar (and possibly, in an arid environment like Santa Rosa at the height of the dry season, of water [41]).

We suggest that when a plant invests in large nectar volumes it is more likely to protect that investment. Estimates for the costs of floral nectar production vary between species and depend upon other factors determining energy expenditure [42], [43], [44], [45], [46] but are probably high enough to “matter” to the plant. This view is reinforced as some plant species recoup part of the costs by reabsorbing nectar, which not only helps to maintain a stable concentration and volume of nectar [47] but also allows the recycling of unused resources after pollination, and their re-direction towards seed production [48], [49], [50]. As demonstrated by Junker *et al*. [34] in Hawaii, when they are not needed floral defences against ant nectar-thieves are convergently lost by native plants. Future studies may link whole plant nectar costs with ant-repellence.

Pollen-based ant-repellence appears to be the commonest method used by angiosperms to repel ant-visitation, since most tests to date have localised the repellence to freshly dehiscing flowers, to anthers, to polyads, or to pollen grains [27], [29], [30]. This repellence is most effective at close range, in a similar way to the silk of some orb-web spiders being repellent to ants only upon contact [51]. As most of the species studied produced flowers that lasted less than a day, pollen-based repellence would be sufficient to dissuade ant-recruitment to flowers throughout the most crucial period of pollinator visitation, leaving only a short period of vulnerability in protandrous species where the stigma was receptive but no further self-pollen was present. In our tactile trials it was impossible to discern the exact nature of repellence from fresh flowers or anthers: whether it operates through VOCs in sufficiently high concentrations at close range, or through direct antennal contact. As Raguso [52] pointed out, insect interactions with flowers at close range “blur the distinction between olfaction, gustation and contact chemoreception as modes of action for chemical floral features”. From a practical perspective, and from the perspective of the ants involved, there is little functional difference between the two modes. However, understanding the mechanisms involved would be of interest in identifying selective actions on floral traits.

Where anthers were not involved, repellence came from trichomes on the inflorescences of *Ruellia inudata* and *Merremia aegyptia*, though only to smaller ant species, suggesting that they provide purely mechanical defence (no glandular trichomes were observed). *Stachytarpheta* species elicited repellence when ant antennae came into contact with the upper petal surfaces (cf., the ant-repellent petals of *Ferocactus wislizeni* [1]), and this triggered agitated antennal grooming without contact with anthers or pollen. At no time was repellent nectar observed. Instead, ants often fed on nectar that had leaked from the bases of repellent flowers, such as *Malvaviscus arboreus*.

Overall we found some form of floral ant-repellence in approximately a third of species tested in the dry forest environment. This is similar to the proportion reported by Willmer *et al*. [30] in the UK and by Junker *et al*. [31] in the wet forest of Borneo. However, there are some discrepancies between our results and those of Ghazoul [35], who also worked at Santa Rosa on some of the same plant species; e.g., some species that Ghazoul found to be ant-repellent (*Cochlospermum vitifolium*, *Ipomoea trifida* and *Gliricidia sepium*) provoked no responses from ants in our trials. The 2 acacia mutualist ant species used by Ghazoul were possible reacting to unfamiliar, non-host, scents. The responses of the more generalist C. novograndensis, may better reflect how common nectar thieving ants interact with flowers.

### b) Relationship between other plant and floral traits and ant-repellence

While the likelihood of floral ant-repellence appears to be linked with nectar volume, accessibility to nectar may also be taken into account. In many cases accessibility will be more strongly influenced by other selection pressures on floral evolution, especially selection for the most efficient pollinator. Selection to prevent nectar theft by ants will either be complimentary to existing floral traits, as in the case of the narrow-flowered hawkmoth-pollinated *Randia monantha,* or will act separately from floral morphology, as in the open bat-pollinated *Crescentia cujete*. This contrasts with the consistent trade-off of VOC repellence and physical protection identified by Willmer *et al*. [30] and Junker *et al*. [34]. Out of the 18 species studied by Junker *et al*. [31] access to nectar was not a predictive factor for ant-repellence, but as nectar volume was not reported we cannot say if this was correlated with ant-repellence. The one species tested that did not produce nectar, *Diospyros durionoides* (Ebenaceae), was not significantly ant-repellent.

Whether or not a plant species develops ant-repellent traits may also depend on its degree of self-compatibility, and the likelihood and costs of geitonogamy. Nectar theft may often lead to reduced seed set, favouring ant-repellence; but lack of such repellence may be more favoured where visitation by ants promotes outcrossing by making pollinators move on more frequently, increasing pollen dispersion [53], [54]. It is possible that other species cannot produce ant-repellence, either at the level of biochemical pathways or because repellence may have too great a disruptive influence on legitimate floral visitors. Conversely, the presence of certain floral traits, such as essential oil glands, may increase the likelihood of ant-repellence in some lineages.

While the proportion of ant-repellence appeared to differ significantly between plant families the effect was reduced as coverage within families increased. Related plant species were usually similarly repellent only when morphologically similar, indicating a stronger link between repellence and pollination syndrome than repellence and phylogeny. Thus, within the Malvaceae tested, the hummingbird-pollinated shrubs *Malvaviscus arboreus* and *Helictores guazumaefolia* possess very similar floral structure and were both repellent to *C. novograndensis* (though different repellent components must be involved since *Ectatomma ruidum* was repelled only by *M.* arboreus); but the open-flowered *Pavonia cancellata* (also Malvaceae) had no such repellent traits. Similarly within the Fabales a clear contrast existed between the ant-repellent Mimosoideae (*Acacia collinsii*, *Bauhinia ungulata*, *Enterolobium cyclocarpu*), and the non-repellent Faboideae (*Centrosema pulmeri*, *Desmodium* sp, *Gliricidia sepium*, *Haemotoxylum brasiletto*, *Securidaca sylvestris*). Ant-repellence is common within the American and African *Acacia* [28], [30] but it may also be common in related genera within the mimosoid subfamily, which usually have highly exposed anthers giving easy access to pollen. Within the Faboideae tested (with the exception of *Haemotoxylum brasiletto*) the pollen is protected behind keel petals, and anthers are often spring-loaded to cover the first visitor with pollen. None of the species with enclosed anthers had repellent pollen, which provides further (indirect) evidence that repellence may be adaptive and dependent on the nature of nectar and pollen rewards provided.

### c) Ant-repellence effectiveness against different ant species

In some plant species ant-repellent traits had a broad effectiveness against a variety of ant species, e.g., *Stachytarpheta jamaicensis*, *Malvaviscus arboreus*, and *Cordia alliodora* flowers were all effectively repellent against multiple ant species, to varying degrees. In contrast, the effect of *Barleria oenotheroides* pollen and *Ruellia inudata* trichomes was restricted to a small number of ant species.

The large predatory ant *Ectatomma ruidum* was only influenced by the floral traits of two of the many species that were repellent to *Camponotus novograndensis*. Therefore there may be little selective pressure to protect potential pollinators from direct ant predation. Ants' impact on pollinator populations is still poorly understood, but perhaps the threat they pose, in comparison to more efficient crab spiders [55] or competing bees [56], is too small to influence floral evolution. One exception may be *Acacia collinsii*, where repellence is likely to have evolved for pollinator protection. While *A. collinsii*'s ant-repellence is not effective against *E. ruidum* it does trigger a strong response from *C. novograndensis*, though this species rarely comes into contact with *A. collinsii* inflorescences and poses no threat to potential pollinators. This response in a non-mutualist is probably a by-product of selection to ensure that ant-guards do not interfere with pollination. No effect of *A. collinsii* VOCs was detected in our scent trials, suggesting that floral its VOCs are very limited in range. The other ant-plant used here, *C. alliodora,* is inhabited by ants with very low aggression posing no threat to pollinators, so its repellence probably arose once again to prevent nectar theft.

### d) Conclusions

This study provides further groundwork for our understanding of how ants and flowers interact. It highlights the importance of potential constraints on floral evolution imposed by recruitable ants acting as nectar-thieves, and the complexity of floral characteristics that together attract potential mutualistic flower visitors and defend against exploitative visitors.

While several different types of ant-repelling traits have been identified, by far the most common is ant-repellent pollen. Discovering the range of chemicals involved in this repellence will be crucial not just for understanding how it arose but also in understanding how ants interpret varied chemical signals from their environment, especially those similar to ant pheromones.

The positive correlation between floral ant-repellence and nectar volume suggests that to understand the role of ant nectar-thieves in floral evolution further information about the costs of nectar production is essential. Conversely, defence of pollinators against aggressive ants may be rather unimportant with no particular repellence of large predatory ant species. Given that interactions with other animals, such as pollinators and herbivores, are strongly selective on floral traits it is interesting that less recognised interactions with nectar-thieves could produce a significant trend in floral ant-repellence.

## Supporting Information

Table S1 Plant species tested against *Camponotus novograndensis* in tactile trials. K-W tests were for differences between agitated responses in ants from fresh flowers and other floral part used (older, pollen and nectar depleted flowers in most cases) with significant differences shown in bold. Mode of pollinations was determined from visits observed while flowers were selected and peak dehiscence time determined combined with literature searches for each species. N = number of ants used in tactile trials.
(DOCX)

## References

[1] Ness JH (2006) A mutualism's indirect costs: the most aggressive plant bodyguards also deter pollinators. Oikos 113: 506–514.

[2] Lach L (2008a) Argentine ants displace floral arthropods in a biodiversity hotspot. Divers Distrib 14: 281–290.

[3] Hansen DM, Müller CB (2009) Invasive ants gecko pollination and seed dispersal of the endangered plant *Roussea simplex* in Mauritius. Biotropica 41: 202–208.

[4] Oliver PS, Cook JM, Leather SR (2007) When are ant-attractant devices a worthwhile investment? *Vica faba* extrafloral nectaries and *Lasius niger* ants. Popul Ecol 49: 265–273.

[5] Styrsky JD, Eubanks MD (2007) Ecological consequences of interactions between ants and honeydew-producing insects. Proc R Soc Lond Ser B: Biol Sci 274: 151–164. 10.1098/rspb.2006.3701 PMC1685857 17148245

[6] Hodson AK, Gastreich KR (2006) Evidence for a novel mutualism in the tropical understory shrub *Piper urostachyum* . Biotropica 38: 127–131.

[7] Schemske DW (1980) The evolutionary significance of extrafloral nectar production by *Costus woodsonii* (Zingiberaceae): an experimental analysis of ant protection. J Ecol 68: 959–967.

[8] Apple JH, Feener DH (2001) Ant visitation of extrafloral nectaries of *Passiflora*: the effects of nectary attributes and ant behaviour on patterns in facultative ant-plant mutualisms. Oecologia 127: 409–416. 28547111 10.1007/s004420000605

[9] Davidson DW (1997) The role of resource imbalances in the evolutionary ecology of tropical arboreal ants. Biol J Linn Soc 61: 153–181.

[10] Blüthgen N, Verhaagh M, Goitia W, Jeffé K, Morawetz W, Barthlott W (2000) How plants shape the ant community in the Amazonian rainforest canopy: the key role of extrafloral nectaries and homopteran honeydew. Oecologia 125: 229–240. 24595834 10.1007/s004420000449

[11] Tillberg CV (2004) Friend or foe? A behavioural and stable isotopic investigation of an ant-plant symbiosis. Oecologia 140: 506–515. 15179580 10.1007/s00442-004-1601-8

[12] Janzen DH (1966) Coevolution of mutualism between ants and acacias in Central America. Evolution 20: 249–275. 28562970 10.1111/j.1558-5646.1966.tb03364.x

[13] Stanton ML, Palmer TM, Young TP (2002) Competition-colonization trade-offs in a guild of African acacia-ants. Ecol Monogr 72: 347–363.

[14] Heil M, McKey D (2003) Protective ant-plant interactions as model systems in ecological and evolutionary research. Annu Rev Ecol Evol Syst 34: 425–453.

[15] Suarez AV, Moraes C, Ippolito A (1997) Defense of *Acacia collinsii* by an obligate and nonobligate ant species: the significance of encroaching vegetation. Biotropica 30: 480–482.

[16] Sagers CL, Ginger SM, Evans RD (2000) Carbon and nitrogen isotopes trace nutrient exchange in an ant-plant mutualism. Oecologia 123: 582–586. 28308767 10.1007/PL00008863

[17] Beattie AJ, Turnbull C, Hough T, Jobson S, Knox B (1985) The vulnerability of pollen and fungal spores to ant secretions: evidence and some evolutionary implications. Am J Bot 72: 606–614.

[18] Vega C, Arista M, Ortiz PL, Herrera CM, Talavera S (2009) The ant-pollination system of *Cytinus hypcistis* (Cytinaceae), a Mediterranean root holoparasite. Ann Bot 103: 1065–1075. 19258337 10.1093/aob/mcp049 PMC2707910

[19] Altshuler DL (1999) Novel interactions of non-pollinating ants with pollinators and fruit consumers in a tropical forest. Oecologia 119: 600–606. 28307720 10.1007/s004420050825

[20] Lach L (2008b) Floral visitation patterns of two invasive ant species and their effects on other hymenopteran visitors. Ecol Entomol 33: 155–160.

[21] Lennartsson T (2002) Extinction thresholds and disrupted plant-pollinator interactions in fragmented plant populations. Ecology 83: 3060–3072.

[22] Galen C, Butchart B (2003) Ants in your plants: effects of nectar-thieves on pollen fertility and seed-siring capacity in the alpine wildflower, *Polemonium viscosum* . Oikos 101: 521–528.

[23] Blancafort X, Gómez C (2005) Consequences of the Argentine ant, *Linepithema humile* (Mayr), invasion on pollination of *Euphorbia characias* (L.) (Euphorbiaceae). Acta Oecolgica 28: 49–55.

[24] Guerrant EO, Fiedler PL (1981) Flower defences against nectar-pilferage by ants. Biotropica 13: 25–33.

[25] Galen C, Kaczorowski R, Todd SL, Geib J, Raguso RA (2011) Dosage-dependent impacts of a floral volatile compound on pollinators, larcenists, and the potential for floral evolution in the alpine skypilot *Polemonium viscosum* . Am Nat 177: 258–272. 21460561 10.1086/657993

[26] Junker RR, Blüthgen N (2010) Floral scents repel facultative flower visitors, but attract obligate ones. Ann Bot 105: 777–782. 20228087 10.1093/aob/mcq045 PMC2859918

[27] Willmer PG, Stone GN (1997) How aggressive ant-guards assist seed-set in *Acacia* flowers. Nature 388: 165–167.

[28] Raine NE, Pierson AS, Stone GN (2007) Plant-pollinator interactions in a Mexican *Acacia* community. Arthropod-Plant Interact 1: 101–117.

[29] Nicklen EF, Wagner D (2006) Conflict resolution in an ant-plant interaction: *Acacia constricta* traits reduce ant costs to reproduction. Oecologia 148: 81–87. 16450177 10.1007/s00442-006-0359-6

[30] Wilmer PG, Nuttman CV, Raine NE, Stone GN, Pattrick JG, et al (2009) Floral volatiles controlling ant behaviour. Funct Ecol 23: 888–900.

[31] Junker RR, Chung AYC, Blüthgen N (2007) Interaction between flowers, ants and pollinators: additional evidence for floral repellence against ants. Ecol Res 22: 665–670.

[32] Junker RR, Blüthgen N (2008) Floral scents repel potentially nectar-thieving ants. Evol Ecol Res 10: 295–308.

[33] Agarwal VM, Rastogi N (2008) Role of floral repellents in the regulation of flower visits of extrafloral nectary-visiting ants in an Indian crop plant. Ecol Entomol 33: 59–65.

[34] Junker RR, Daehler CC, Dötterl S, Keller A, Blüthgen N (2011) Hawaiian ant-flower networks: nectar-thieving ants prefer undefended native over introduced plants with floral defences. Ecol Monogr 81: 295–311.

[35] Ghazoul J (2001) Can floral repellents pre-empt potential ant-plant conflicts? Ecol Lett 4: 295–299.

[36] Schatz B, Wcislo WT (1999) Ambush predation by the ponerine ant *Ectatomma ruidum* Roger (Formicidae) on a sweat bee *Lasioglossum umbripenne* (Halictidae), in Panama. J Insect Beh 12: 641–663.

[37] Armbruster WS, Fenster CB, Dudash MR (2000) Pollination “principles” revisited: specialization, pollination syndromes and the evolution of flowers. In The Scandinavian Association for Pollination Ecology Honours Knut Faegri, Mat Naturv Klasse Skrifter Ny Serie 39: 179–200.

[38] Ollerton J, Killick A, Lamborn E, Watts S, Whiston M (2007) Multiple meanings and modes: on the many ways to be a generalist flowers. Taxon 56: 717–728.

[39] Willmer PG (2011) Pollination and Floral Ecology. Princeton University Press.

[40] Opler PA (1983) Nectar production in a tropical ecosystem. In The Biology of Nectaries. Columbia University Press New York.

[41] Willmer PG (1986) Foraging patterns and water balance: problems of optimisation for a xerophilic bee, *Chalicodoma sicula* . J Anim Ecol 55: 941–962.

[42] Southwick EE (1984) Photosynthate allocation to floral nectar: a neglected energy investment. Ecology 65: 1775–1779.

[43] Pyke GH (1991) What does it cost a plant to produce floral nectar? Nature 350: 58–59.

[44] Golubov J, Mandujano MC, Montaña C, López-Portillo J, Eguiarte LE (2004) The demographic costs of nectar production in the desert perennial *Prosopis glandulosa* (Mimosoideae): a modular approach. Plant Ecol 170: 267–275.

[45] Leiss KA, Vrieling K, Klinkhamer PGL (2004) Heritability of nectar production in *Echium vulgare* . Heredity 92: 446–451. 15026780 10.1038/sj.hdy.6800439

[46] Ordano M, Ornelas JF (2005) The cost of nectar replenishment in two epiphytic bromeliads. J Trop Ecol 21: 541–547.

[47] Nicolson SW (1995) Direct demonstration of nectar reaborption in the flowers of *Grevillea robusta* (Proteaceae). Funct Ecol 9: 584–588.

[48] Koopowitz H, Marchant TA (1998) Postpollination nectar reabsorption in the African epiphyte *erangis verdickii* (Orchidaceae). Am J Bot 85: 508–512. 21684933

[49] Luyt R, Johnson SD (2002) Postpollination nectar reabsorption and its implications for fruit quality in an epiphytic orchid. Biotropica 34: 442–446.

[50] Nepi M, Stpiczyñska M (2008) Nectar Resorption and Translocation in *Cucurbita pepo* L. and *Platanthera chlorantha* Custer (Rchb.). Plant Biol 9: 93–100. 10.1055/s-2006-924287 16883483

[51] Zhang S, Koh TH, Seah WK, Lai YH, Elgar MA, et al (2012) A novel property of spider silk: chemical defence against ants. Proc R Soc Lon Ser B: Biol Sci 279: 1824–1830. 10.1098/rspb.2011.2193 PMC3297460 22113027

[52] Raguso RA (2004) Why are some floral nectars scented? Ecology 85: 1486–1494.

[53] Maloof JE, Inouye DW (2000) Are nectar robbers cheaters or mutualists? Ecology 81: 2651–2661.

[54] Irwin RE, Brody AK, Waser NM (2001) The impact of floral larceny on individuals, populations and communities. Oecologia 129: 161–168. 28547611 10.1007/s004420100739

[55] Dukas R, Morse DH (2003) Crab spiders affect flower visitation by bees. Oikos 101: 157–163.

[56] Dworshak K, Blüthgen N (2010) Networks and dominance hierarchies: does interspecific aggression explain flower partitioning among stingless bees? Ecol Entomol 35: 216–225.

