# Supplementary material for: Nectar Theft and Floral Ant-Repellence: A Link between Nectar Volume and Ant-Repellent Traits?
Source: PLoS One. 2012 Aug 29;7(8):e43869. doi: 10.1371/journal.pone.0043869 (PMC3430612; doi:10.1371/journal.pone.0043869)
Supplement: Table S1 — Plant species tested against Camponotus novograndensis in tactile trials. K-W tests were for differences between agitated responses in ants from fresh flowers and other floral part used (older, pollen and nectar depleted flowers in most cases) with significant differences shown in bold. Mode of pollinations was determined from visits observed while flowers were selected and peak dehiscence time determined combined with literature searches for each species. N = number of ants used in tactile trials. (DOCX) [file pone.0043869.s001.docx]

**Supporting Information.** Plant species tested against *Camponotus novograndensis* in tactile trials. K-W tests were for differences between agitated responses in ants from fresh flowers and other floral part used (older, pollen and nectar depleted flowers in most cases) with significant differences shown in bold. Mode of pollinations was determined from visits observed while flowers were selected and peak dehiscence time determined combined with literature searches for each species. N = number of ants used in tactile trials.

| **Family** | **Species** | **Growth Form** | **Nectar Volume** | **K-W χ^2^** | **p-value** | **Mode of Pollination: Common Potential Pollinators** | **N** | **Tested in Scent Trails** |
| --- | --- | --- | --- | --- | --- | --- | --- | --- |
| Acanthaceae | *Aphelandra scabra* | shrub | 3 | 0.198 | 0.656 | Hummingbirds - Specialist | 11 | y |
|  | *Barleria oenotheroides* | herb | 2 | 12.233 | **<0.001** | Bees - Specialist | 14 | y |
|  | *Blechum pyramidatum* | herb | 2 | 2.332 | 0.127 | Bees - Specialist | 11 | n |
|  | *Dyschoriste valeriana* | herb | 2 | 0.819 | 0.365 | Bees - Specialist | 12 | n |
|  | *Justicia carthaginensis* | herb | 2 | 0.495 | 0.482 | Large bees - Specialist | 12 | n |
|  | *Ruellia inudata* | herb | 2 | 0.791 | 0.374 | Bees, butterflies - Generalist | 13 | y |
| Apocynaceae | *Fosteronia spicata* | tree | 1 | 0.006 | 0.939 | Bees, butterflies - Generalist | 9 | y |
|  | *Thevetia peruviana* | tree | 3 | 0.005 | 0.942 | Bees, butterflies - Generalist | 10 | y |
| Asteraceae | *Ageratum conyzoides* | herb | 1 | 0.860 | 0.354 | Bees, butterflies - Generalist | 11 | y |
|  | *Lasianthaea fructicosa* | herb | 1 | 0.002 | 0.965 | Bees, butterflies - Generalist | 15 | y |
|  | *Melanthera aspera* | herb | 1 | 15.016 | **<0.001** | Bees, butterflies - Generalist | 12 | y |
| Bignoniaceae | *Crescentia cujete* | tree | 4 | 16.091 | **<0.001** | Bats - Specialist | 9 | y |
|  | *Tabebuia ochracea* | tree | 2 | 2.692 | 0.260 | Bees - Specialist | 12 | n |
|  | unidentified liana | climber | 2 | 5.846 | 0.016 | Bees - Specialist | 11 | n |
| Boraginaceae | *Cordia alliodora* | tree | 1 | 16.178 | **<0.001** | Bees, butterflies - Generalist | 21 | y |
|  | *Cordia guanacastensis* | shrub | 1 | 6.302 | 0.012 | Bees, butterflies, muscids - Generalist | 13 | y |
| Bromeliaceae | *Bromelia pinguin* | shrub | 0 | 1.585 | 0.453 | Bees - Specialist | 12 | y |
| Capparidaceae | *Capparis indica* | shrub | 2 | 13.91 | **<0.001** | Bat, hawkmoth - Specialist | 12 | y |
| Caricaceae | *Carica papaya* | tree | 4 | 5.241 | 0.022 | Long-tongued bees, lepidopterans - Specialist | 17 | y |
| Cochlospermaceae | *Cochlospermum vitifolium* | tree | 0 | 1.182 | 0.277 | Buzz pollinated by bees - Generalist | 16 | y |
| Convolvulaceae | *Ipomoea trifida* | climber | 2 | 1.426 | 0.232 | Bees, butterflies, flies - Generalist | 16 | y |
|  | *Merremia aegyptia* | climber | 2 | 8.121 | 0.004 | Bees, butterflies, flies - Generalist | 12 | n |
| Cucurbitaceae | unidentified dioecious liana (male) | climber | 1 | 3.300 | 0.069 | Bees, flies - Generalist | 11 | n |
| Fabaceae | *Acacia collinsii* | tree | 0 | 23.875 | **<0.001** | Bees - Specialist | 12 | y |
|  | *Bauhinia ungulata* | shrub | 4 | 7.430 | **<0.001** | Bats - Specialist | 12 | y |
|  | *Centrosema plumeri* | climber | 0 | 2.156 | 0.142 | Large bees - Specialist | 11 | n |
|  | *Desmodium* sp. | herb | 0 | 0.200 | 0.655 | Small bess - Specialist | 11 | n |
|  | *Enterolobium cyclocarpum* | tree | 1 | 25.878 | **<0.001** | Moths, beetles - Generalist | 10 | y |
|  | *Gliricidia sepium* | tree | 0 | 5.840 | 0.054 | Medium-sized bees - Specialist | 11 | y |
|  | *Haematoxylum brasiletto* | tree | 0 | 0.030 | 0.859 | Bees - Specialist | 12 | n |
|  | *Securidaca sylvestris* | tree | 0 | 1.138 | 0.566 | Medium-large bees - Specialist | 11 | n |
| Flacourtiaceae | *Casearia corymbosa* | shrub | 1 | 6.111 | **<0.001** | Bees, flies - Generalist | 9 | y |
| Malpighiaceae | *Byrsonima crassifolia* | tree | 0 | 0.328 | 0.849 | Oil-collecting bees - Specialist | 11 | y |
| Malvaceae | *Helictores guazumifolia* | shrub | 4 | 7.500 | **<0.001** | Hummingbirds - Specialist | 11 | y |
|  | *Malvaviscus arboreus* | shrub | 4 | 10.66 | **<0.001** | Hummingbirds - Specialist | 15 | y |
|  | *Pavonia cancellata* | climber | 2 | 0.543 | 0.461 | Bees, butterflies - Generalist | 12 | n |
| Oxalidaceae | *Oxalis frutescens* | herb | 1 | 6.374 | **<0.001** | Small bees, flies - Generalist | 19 | y |
| Rosaceae | *Muntingia calabura* | tree | 1 | 0.035 | 0.852 | Small bees, flies - Generalist | 12 | y |
| Rubiaceae | *Randia monantha* | tree | 4 | 1.007 | 0.316 | Hawkmoths - Specialist | 12 | y |
| Scrophulariaceae | unidentified herb | herb | 1 | 2.133 | 0.144 | Small bees, flies - Generalist | 8 | n |
| Simaroubaceae | *Simarouba glauca* | tree | 1 | 2.560 | 0.110 | Bees, flies - Generalist | 12 | n |
| Sapindaceae | *Paullinia cururu* | climber | 1 | 0.946 | 0.623 | Bees, flies - Generalist | 12 | y |
| Solanaceae | *Solanum rugosum* | shrub | 0 | 1.839 | 0.175 | Buzz pollinated by bees - Generalist | 12 | y |
| Sterculiaceae | *Guazuma ulmifolia* | tree | 1 | 1.643 | 0.200 | Bees, flies - Generalist | 12 | y |
| Theophrastaceae | *Jaquinia pungens* | shrub | 1 | 3.866 | 0.145 | Hummingbirds? - Specialist | 8 | y |
| Turneraceae | *Turnera pumila* | herb | 1 | 0.024 | 0.877 | Bees, butterflies, flies - Generalist | 11 | n |
| Verbenaceae | *Cornutia grandiflora* | shrub | 0 | 0.022 | 0.882 | Buzz pollinated by bees - Generalist | 11 | y |
|  | *Lantana camera* | shrub | 1 | 2.890 | 0.089 | Hummingbirds, butterflies - Specialist | 12 | y |
|  | *Stachytarpheta jamaicensis* | herb | 3 | 7.411 | **<0.001** | Bees, butterflies - Generalist | 12 | n |
